# The Antiarrhythmic and Hypotensive Effects of S-61 and S-73, the Pyrrolidin-2-one Derivatives with α_1_-Adrenolytic Properties

**DOI:** 10.3390/ijms231810381

**Published:** 2022-09-08

**Authors:** Klaudia Lustyk, Kinga Sałaciak, Agata Siwek, Jacek Sapa, Paula Zaręba, Adam Gałuszka, Karolina Pytka

**Affiliations:** 1 Department of Pharmacodynamics, Faculty of Pharmacy, Jagiellonian University Medical College, Medyczna 9, 30-688 Krakow, Poland; 2 Department of Pharmacobiology, Faculty of Pharmacy, Jagiellonian University Medical College, Medyczna 9, 30-688 Krakow, Poland; 3 Department of Physiochemical Drug Analysis, Faculty of Pharmacy, Jagiellonian University Medical College, Medyczna 9, 30-688 Krakow, Poland; 4 Department of Automatic Control and Robotics, Silesian University of Technology, Akademicka 2A, 44-100 Gliwice, Poland

**Keywords:** alpha-1 adrenergic receptor, alpha-1 adrenolytics, pyrrolidin-2-one, arrhythmia, adrenaline-induced arrhythmia

## Abstract

Heart rhythm abnormalities are a cause of many deaths worldwide. Unfortunately, the available antiarrhythmic drugs show limited efficacy and proarrhythmic potential. Thus, efforts should be made to search for new, more effective, and safer pharmacotherapies. Several studies suggested that blocking the α_1_-adrenoceptors could restore normal heart rhythm in arrhythmia. In this study, we aimed to assess the antiarrhythmic potential of S-61 and S-73, two novel pyrrolidin-2-one derivatives with high affinity for α_1_-adrenergic receptors. First, using radioligand binding studies, we demonstrated that S-61 and S-73 did not bind with β_1_-adrenoceptors. Next, we assessed whether S-61 and S-73 could protect rats against arrhythmia in adrenaline-, calcium chloride- and aconitine-induced arrhythmia models. Both compounds showed potent prophylactic antiarrhythmic properties in the adrenaline-induced arrhythmia model, but the effect of S-61 was more pronounced. None of the compounds displayed antiarrhythmic effects in calcium chloride- or aconitine-induced arrhythmia models. Interestingly, both derivatives revealed therapeutic antiarrhythmic activity in the adrenaline-induced arrhythmia, diminishing heart rhythm irregularities. Neither S-61 nor S-73 showed proarrhythmic potential in rats. Finally, the compounds decreased blood pressure in rodents. The hypotensive effects were not observed after coadministration with methoxamine, which suggests the α_1_-adrenolytic properties of both compounds. Our results confirm that pyrrolidin-2-one derivatives possess potent antiarrhythmic properties. Given the promising results of our experiments, further studies on pyrrolidin-2-one derivatives might result in the development of a new class of antiarrhythmic drugs.

## 1. Introduction

Sudden cardiac death is one of the leading causes of mortality in developed countries and accounts for 50% of all cardiovascular deaths and around 15–20% of all deaths [1,2]. The principal cause of sudden death are malignant cardiac arrhythmias, which lead to the lack of blood supply to vital organs like the brain [3]. Unfortunately, the available antiarrhythmic drugs, which either block a specific ionic current, multiple ionic channels, or suppress β-adrenergic signaling, are often ineffective and possess serious side effects, such as proarrhythmic potential [4,5,6]. Therefore, there is an urgent need to develop novel drugs which could safely and effectively treat life-threatening heart rhythm disturbances.

Studies demonstrated that α_1_-adrenoceptor might play an essential role in heart rhythm disturbances [7]. On the one hand, the receptor is vital for heart functioning, i.e., mediating physiologic or adaptive hypertrophy [8] or augmenting contractility [9,10,11]. On the other hand, the α_1_-adrenergic receptors may be involved in the etiology of the ischemia- and reperfusion-related arrhythmias [12,13]. What further supports the potential of α_1_-adrenergic receptors as biological targets to treat heart rhythm abnormalities is that α_1_-adrenolytics showed antiarrhythmic properties in animal models of ischemia-induced arrhythmias [14,15,16,17,18]. Therefore, considering the above premises, it is worth searching for new antiarrhythmic drugs among α_1_ receptor antagonists.

Pyrrolidin-2-one derivatives are a group of compounds that are proven not only to bind strongly to the α_1_-adrenergic receptor but also show antiarrhythmic properties in animal models [19,20]. Our former studies on S-75, a pyrrolidin-2-one derivative, demonstrated its α_1_-adrenolytic properties and significant antiarrhythmic activity in rat arrhythmia models [19]. Given the promising results of our previous experiments in search of compounds with more favorable pharmacological properties, this study aimed to investigate the antiarrhythmic potential of two new pyrrolidin-2-one derivatives, which have shown high affinity for α_1_-adrenoceptors in animal models of arrhythmia.

## 2. Results

### 2.1. S-61 and S-73 Did Not Show Affinity for β_1_-Adrenergic Receptors

Both S-61 and S-73 did not bind to β_1_-adrenergic receptors (Table 1).

### 2.2. S-61 and S-73 Showed Prophylactic Antiarrhythmic Activity in Arrhythmia Models Induced by Adrenaline, but Not by Calcium Chloride and Aconitine

Adrenaline (20 μg/kg), calcium chloride (140 mg/kg), or aconitine (20 μg/kg) administered intravenously elicited heart rhythm irregularities, leading to the animals’ death (Table 2).

S-61 administered at a dose of 0.5 mg/kg before the arrhythmogen (adrenaline) diminished the number of heart rhythm disturbances, such as extrasystoles, conduction blocks, and bradycardia, protecting animals from death (ED_50_ = 0.2 (0.11–0.36)). In addition, S-73 at a dose of 0.5 mg/kg decreased the amount of adrenaline-induced extrasystoles, atrioventricular blocks, bradycardia, and animal mortality (ED_50_ = 0.36 (0.24–0.53)).

Neither S-61 nor S-73 minimized the number of calcium chloride- and aconitine-induced heart rhythm irregularities, showing no activity in these arrhythmia models.

### 2.3. S-61 and S-73 Showed Therapeutic Antiarrhythmic Activity in Adrenaline-Induced Arrhythmia Model

**Figure 1:**
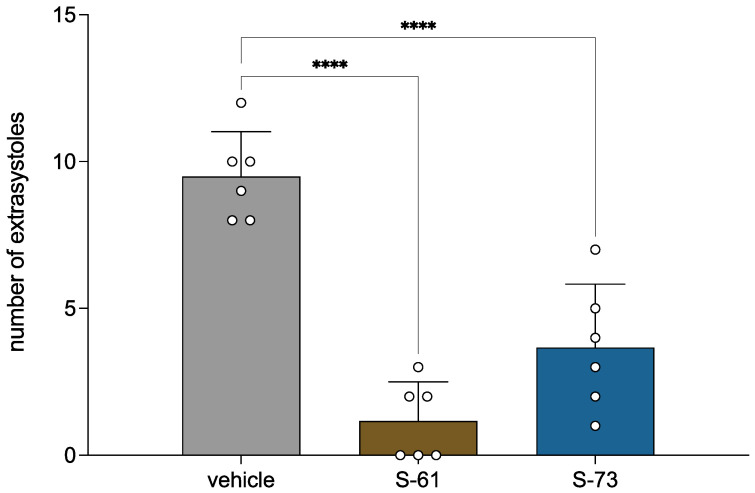
The therapeutic antiarrhythmic activity of S-61 and S-73 in the adrenaline-induced arrhythmia model. After an intravenous (*iv*) injection of adrenaline (20 µg/kg), the tested compounds were immediately administered *iv* at a dose of 5 mg/kg. The control group received no additional treatment. The ECG was recorded for the first 2 min and then at the 5th, 10th, and 15th min of the experiment. The criterion of antiarrhythmic activity was the decrease or complete absence of extrasystoles in the ECG recording compared with the control group. The results are presented as means ± SD. Statistical analysis: one-way ANOVA (Dunnett’s post hoc); **** *p* < 0.0001; n = 6 rats.

Both S-61 and S-73 significantly decreased the number of arrhythmogen-induced extrasystoles by 87.7% and 61.4%, respectively, compared to the vehicle-treated group (F(2,15) = 37.69, *p* < 0.0001, Figure 1).

The tested compounds also decreased the occurrence of bradycardia and conduction blocks in adrenaline-treated rats (Table 3).

### 2.4. S-61 and S-73 Decreased the Heart Rate in the Normal ECG in Rats

S-61 administered at a dose of 5 mg/kg did not affect the PQ (F(3,15) = 6.698, *p* < 0.01), QRS (F(3,15) = 2.2, ns), QT_c_ (F(3,15) = 2.657, ns), or QT (F(3,15) = 1.253, ns) (Table 4). However, the tested compound decreased the heart rate by 12.9% and 10.6%, 5 and 10 min after the injection, respectively (F(3,15) = 63.4, *p* < 0.0001). 

After administration of S-73 at a dose of 5 mg/kg, there were no statistically significant changes regarding PQ (F(3,15) = 2.313, ns), QRS (F(3,15) = 2.358, ns), QT_c_ (F(3,15) = 2.496, ns), or QT (F(3,15) = 1.253, ns), but this compound decreased the heart rate by 13.1%, 13.4%, and 15.9% in the 5th, 10th, and 15th min of ECG recording, respectively (F(3,15) = 28.87, *p* < 0.0001) (Table 4).

### 2.5. S-61 and S-73 Decreased the Blood Pressure in the Normotensive Rats

**Figure 2:**
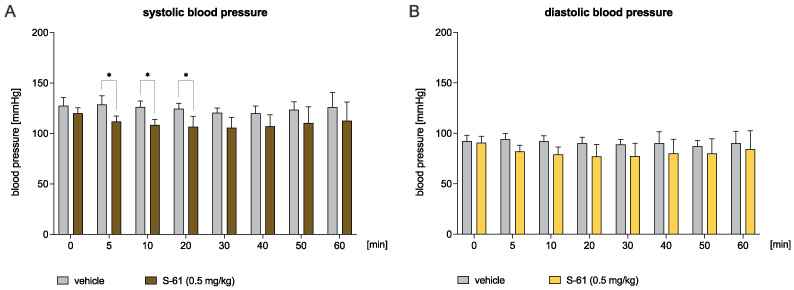
The effect of S-61 on the systolic (Panel **A**) and diastolic blood pressure (Panel **B**) in the normotensive rats. The blood pressure was measured before and 5, 10, 20, 30, 40, 50, and 60 min after the intravenous (*iv*) administration of either S-61 at a dose of 0.5 mg/kg or vehicle (saline). The results are presented as means ± SD. Statistical analysis: two-way repeated measures ANOVA (Bonferroni post hoc); * *p* < 0.05; n = 6 rats.

The hypotensive effect of S-61 given at a dose of 0.5 mg/kg was present in the 5th, 10th, and 20th min post injection—the compound decreased the systolic blood pressure by 13.0%, 14.0%, and 12.3%, respectively (Time: F(7,70) = 3.284, *p* < 0.01; Treatment: F(1,10) = 10.41, *p* < 0.01; Time × Treatment: F(7,70) = 0.7296, ns; Figure 2A). S-61 did not influence the diastolic blood pressure in the normotensive rats (Time: F(7,70) = 1.818, ns; Treatment: F(1,10) = 4.614, ns; Time × Treatment: F(7,70) = 0.9938, ns; Figure 2B).

**Figure 3:**
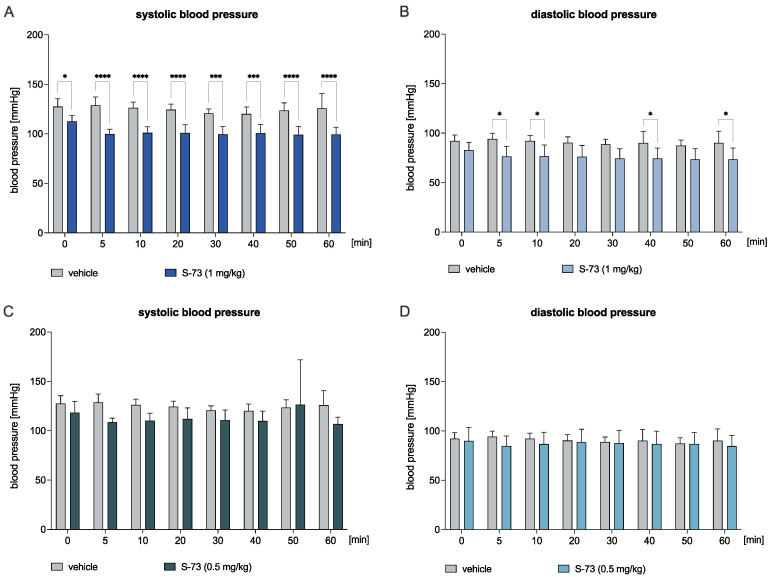
The effect of S-73 on the systolic (Panel **A**,**C**) and diastolic blood pressure (Panel **B**,**D**) in the normotensive rats. The blood pressure was measured before and 5, 10, 20, 30, 40, 50, and 60 min after the intravenous (*iv*) administration of either S-73 at the dose of 1 and 0.5 mg/kg or vehicle (saline). The results are presented as means ± SD. Statistical analysis: two-way repeated measures ANOVA (Bonferroni post hoc); * *p* < 0.05, *** *p* < 0.001, **** *p* < 0.0001; n = 6 rats.

The administration of S-73 at a dose of 1 mg/kg decreased both systolic (Time: F(7,70) = 4.315, *p* < 0.001; Treatment: F(1,10) = 43.52, *p* < 0.0001; Time × Treatment: F(7,70) = 2.076, ns; Figure 3A) and diastolic blood pressure (Time: F(7,70) = 2.062, ns; Treatment: F(1,10) = 11.50, *p* < 0.01; Time × Treatment: F(7,70) = 0.6238, ns; Figure 3B). We observed a decrease in the systolic blood pressure by 11.6–22.4% from 0 to 60 min after the compound’s injection. Moreover, the diastolic blood pressure was decreased in the 5th, 10th, 40th, and 60th min by 18.8%, 16.8%, 17.6%, and 18.5%, respectively, after the compound’s administration. On the other hand, S-73 administered at a dose of 0.5 mg/kg did not significantly affect the systolic (Time: F(7,70) = 1.185, ns; Treatment: F(1,10) = 4.59, ns; Time × Treatment: F(7,70) = 1.258, ns; Figure 3C) and diastolic blood pressure (Time: F(7,70) = 0.6734, ns; Treatment: F(1,10) = 0.5188, ns; Time × Treatment: F(7,70) = 0.8747, ns; Figure 3D). 

### 2.6. S-61 and S-73 Abolished the Pressic Effect of Methoxamine

**Figure 4:**
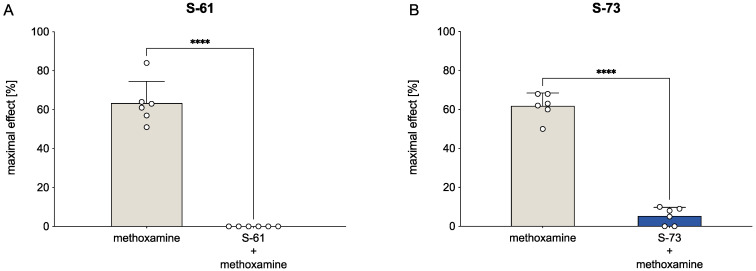
The effect of S-61 (Panel **A**) and S-73 (panel **B**) on blood pressure response to methoxamine. Pressor response to methoxamine (150 μg/kg) was estimated before and 5 min after the intravenous (*iv*) administration of both compounds at a 1 mg/kg dose. The results are presented as means ± SD. Statistical analysis: paired *t*-test; **** *p* < 0.0001; n = 6 rats.

S-61 administered at a dose of 1 mg/kg attenuated the methoxamine pressor response by 100% (t(5) = 13.87, *p* < 0.0001, Figure 4). The pressor effect of methoxamine was also abolished after the injection of the S-73 at a dose of 1 mg/kg by 91.4% (t(5) = 17.05, *p* < 0.0001).

## 3. Discussion

In the present paper, we discovered that S-61 and S-73, pyrrolidine-2-one derivatives, expose prophylactic and therapeutic antiarrhythmic activity in rodents, most probably by targeting α_1_-adrenoceptors. Notably, both compounds did not prolong the QT_c_ interval, so we can assume they do not have torsadogenic potential. Besides, the hypotensive activity of S-61 and S-73 relates to their antagonistic effects toward α_1_-adrenergic receptors.

Many studies on pyrrolidine-2-one derivatives prove their diverse pharmacological effects, such as procognitive [23], anticonvulsant [24], antidepressant [25,26], sleep-promoting [27], anti-inflammatory [28], anticancer [29,30], antifungal [31], antimalarial [32], hypotensive, and antiarrhythmic activity [33]. Furthermore, scientists have discovered that the compounds containing pyrrolidine-2-one fragment often exhibit α-adrenolytic properties [20,34]. Continuing our previous research, we further investigated the effects of two pyrrolidine-2-one derivatives, S-61, and S-73, on the cardiovascular system using rodent models. Experimental rodent models still play an essential role and are extensively used in research on cardiovascular diseases. Animal models not only effectively imitate human cardiovascular disorders, but they can also be easily reproduced and allow the detection of pathological changes.Rats are commonly employed to study the pathophysiology of cardiovascular diseases (e.g., myocardial infarction, hypertension, heart failure, or arrhythmia), as well as for drug development of human diseases.

Our previous study showed that the selected pyrrolidine-2-one derivatives bound strongly to α_1_- (S-61: *p*K_i_ = 7.14; S-73: *p*K_i_ = 6.77) and negligibly to α_2_-adrenergic receptors (S-61: *p*K_i_ = 6.05; S-73: *p*K_i_ = <5) [35]. Moreover, the former experiments revealed their antagonistic properties toward α_1B_-adrenoceptors and no binding with α_1A_-adrenergic receptors [35]. Given the role of α_1B_ receptors in the modulation of cardiac contractility and positive chronotropic effect [36,37], here we evaluated the antiarrhythmic potential of S-61 and S-73.

The sympathetic nervous system plays an essential role in regulating cardiac function. The effects of norepinephrine released from nerve cells innervating the heart are mediated by both α- and β-adrenergic receptors [38]. Considering the effects of the β-adrenergic activation on heart rate or cardiac contractility, as well as the role of β-blockade in the treatment of arrhythmia [39,40], we first investigated the affinity of the compounds for β-adrenoceptors. Our experiments showed that none of the tested compounds demonstrated a significant affinity for β-adrenergic receptors, which excludes their role in the mechanism of the pharmacological effects of S-61 and S-73. This finding aligns with our previous study on another pyrrolidine-2-one derivative, compound S-75 [19].

Several findings indicate a beneficial effect of α_1_-adrenolytics in the pharmacotherapy of arrhythmia [41,42]. There is no doubt that more research on novel antiarrhythmic compounds must be undertaken to overcome the issue of the increasing number of patients suffering from arrhythmia and the lack of effective therapy for this fatal condition. Therefore, in the following step, we verified the potential antiarrhythmic activity of pyrrolidyn-2-one derivatives in models of arrhythmia induced by various chemicals, i.e., adrenaline, calcium chloride, and aconitine. The used arrhythmogens caused arrhythmias manifested by extrasystoles, atrioventricular blocks, bradycardia, fibrillations, and animal mortality. An arrhythmogenic effect of adrenaline results from the interaction with adrenergic receptors. Therefore, we wanted to verify whether S-61 and S-73, which possess a high affinity for α_1_-adrenergic receptors, would be active in this arrhythmia model. The pretreatment with both tested compounds decreased the number of post-adrenaline heart rhythm disturbances indicating their prophylactic antiarrhythmic activity in the adrenaline-induced arrhythmia model. Moreover, calculated ED_50_ value for S-61 was 1.8-fold lower than for carvedilol (ED_50_ = 0.36 mg/kg) [43], suggesting the high importance of α_1_-adrenoceptors blockade for the effectiveness in this model of arrhythmia. Additionally, Kurtzwald-Josefson and collaborators demonstrated that β-blockers lack effectiveness against stress-induced ventricular tachycardia in adult CASQ2Δ/Δ mice, whereas phentolamine—a non-selective α-adrenergic receptor blocker—showed antiarrhythmic activity [38]. Even though α_1_-adrenolytics still have no established role in treating arrhythmia, we strongly highlight that they might be beneficial in ventricular arrhythmias induced by catecholamines, stress, exercises, or ischemia.

Once the prophylactic antiarrhythmic potential of S-61 and S-73 in the adrenaline-induced model of arrhythmia was confirmed, in the next step of our research, we chose calcium chloride- and aconitine-induced rodent models of heart rhythm disturbances. Calcium chloride causes changes in intracellular Ca^2+^ concentration, whereas aconitine modifies intracellular Na^+^ levels leading to arrhythmia. Interestingly, none of the studied compounds protected against heart rhythm disturbances induced by calcium chloride or aconitine, which suggests that S-61 and S-73 do not exert their antiarrhythmic effects via Ca^2+^ or Na^+^ channels.

Our promising results on prophylactic antiarrhythmic activity in adrenaline-induced arrhythmia encouraged us to investigate whether the studied pyrrolidyn-2-one derivatives possess therapeutic antiarrhythmic activity. In this model, pyrrolidine-2-one derivatives were administered right after the intravenous injection of adrenaline to verify their potential to stop the arrhythmia. Our results showed that both compounds quickly restored normal sinus rhythm, reducing the amount of post-arrhythmogen extrasystoles, conduction blocks, bradycardia, and animal mortality. The combination of prophylactic and therapeutic antiarrhythmic activity of the studied pyrroilidin-2-one derivatives indicates their potential to not only prevent attacks of arrhythmia but also to treat them. Such properties of a compound could be of great benefit in the treatment of arrhythmia.

Increasing evidence indicates that the treatment with antiarrhythmic drugs is strongly associated with the occurrence of proarrhythmic ventricular tachyarrhythmias [44]. In particular, drugs that prolong repolarization (i.e., IA and III class of antiarrhythmic drugs) may cause ventricular proarrhythmia in the form of *torsade de pointes* [45]. Therefore, next, we evaluated the influence of S-61 and S-73 on normal ECG in rats. Our experiments revealed, most importantly, that the tested compounds did not prolong the QT interval, so we may assume that they lack torsadogenic potential. Neither PQ interval nor QRS complex were affected by the intravenous administration of S-61 and S-73. The only influenced parameter was the heart rate. S-61 and S-73 elicited negative chronotropic effects but at doses 25- and 14-fold higher than the median effective dose in the adrenaline-induced arrhythmia model. We suspect that this effect may be associated with the blockade of α_1B_-receptors. However, this hypothesis requires further studies.

Knowing that the blockade of α_1_-adrenergic receptors is also responsible for the regulation of blood pressure, next, we evaluated the effect of tested compounds on systolic and diastolic blood pressure after a single intravenous administration in normotensive rats. We found that S-61 and S-73 reduced blood pressure at a dose 2.5- and 2.8-fold higher than the median effective dose in the adrenaline-induced model. The results suggest that lower doses could be used to treat heart rhythm irregularities, whereas higher doses of the tested compounds could benefit patients suffering from both arrhythmia and hypertension.

Finally, we proved that the hypotensive effect of tested pyrrolidine-2-one derivatives was a result of their α_1_-adrenolytic properties. The inhibition of the pressor response to methoxamine, an agonist of α_1_-adrenergic receptors, by the pretreatment with the studied compounds confirmed that finding. The experiment revealed that both S-61 and S-73 decreased the effect induced by methoxamine, suggesting their α_1_-adrenolytic effects.

Our study has some limitations; although the performed experiments showed no antiarrhythmic activity in calcium chloride- and aconitine-induced arrhythmia, we cannot entirely exclude the role of Ca^2+^ and Na^+^ in the molecular mechanism of action. Therefore, further studies are required to indicate the exact cellular mechanism of S-61 and S-73 pharmacological activity. Moreover, their activity in the post-reperfusion model needs investigation to unravel the full antiarrhythmic potential of the studied compounds. Finally, since β_2_ receptors play a role in arrhythmogenesis, we need to investigate the affinity of pyrrolidin-2-ones for β_2_-adrenoceptors.

## 4. Materials and Methods

### 4.1. Drugs

**Figure 5:**
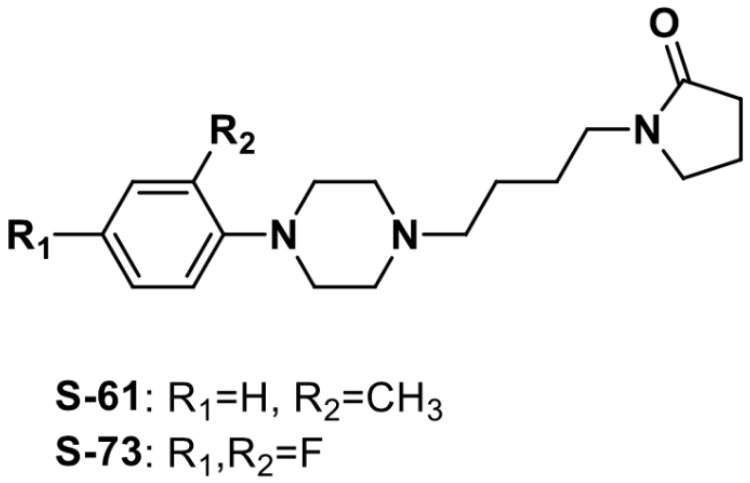
The chemical structure of S-61 and S-73.

The tested compounds: 1-{4-[4-(2-tolyl)piperazin-1-yl]butyl}pyrrolidin-2-one hydrochloride (S-61) and 1-{4-[4-(2,4-difluorophenyl)piperazin-1-yl]butyl} pyrrolidin-2-one hydrochloride (S-73) were synthesized in the Department of Physiochemical Drug Analysis, Faculty of Pharmacy, Jagiellonian University Medical College (Figure 5) [35]. The investigated derivatives were dissolved in saline (Polpharma, Starogard Gdańsk, Poland) and administered intravenously (*iv*).

In the radioligand studies we used propranolol (Sigma-Aldrich, Darmstadt, Germany), dissolved in saline. Commercially available reagents such as adrenaline (Polfa S.A., Warsaw, Poland), and methoxamine (Sigma-Aldrich, Darmstadt, Germany) were dissolved in saline and administered intravenously (*iv*). Thiopental (Sandoz GmgH, Kundl, Austria) was also dissolved in saline and administered intraperitoneally (*ip*). Heparin (Polfa S.A., Warsaw, Poland) was used as an anticoagulant during experiments. The control groups received saline as a vehicle.

### 4.2. Animals

All experiments were performed on male normotensive Wistar rats weighing 200–250 g obtained from an accredited animal house at the Faculty of Pharmacy, Jagiellonian University Medical College, Krakow, Poland. Animals were kept in groups of 3 in plastic cages (42.7 cm × 26.7 cm) in a controlled environment (i.e., ambient temperature (22 ± 2 °C), adequate humidity (40–60%), 12 h light/dark cycle) with ad libitum access to standard pellet food and filtered tap water. Animals were selected randomly for treatment groups; each group consisted of six animals. All experiments were performed between 9:00 and 14:00. Injections were administered in a 1 mL/kg volume. Rats were used only once in each test, and immediately after each experiment, animals were euthanized. Procedures involving animals were conducted according to current European Community and Polish legislation on animal experimentation.

### 4.3. Radioligand Binding Assay

The β_1_-adrenoceptor radioligand binding assay was conducted on the rat cerebral cortex. [^3^H]-CGP-12177 (48 Ci/mmol, β_1_-adrenergic receptor) was used as a specific ligand. The brains were homogenized using ULTRA-TURRAX homogenizer in 10 mL of an ice-cold 50 mM Tris-HCl buffer (pH 7.6). Homogenates were centrifuged at 20,000× *g* for 20 min (0–4 °C). Next, the cell pellet was resuspended in the Tris–HCl buffer and centrifuged again. Radioligand binding assays were performed in plates (MultiScreen/Millipore). The final incubation mixture (volume 300 μL) consisted of 240 μL of the tissue suspension, 30 μL of radioligand solution, and 30 μL of the buffer containing 7–8 concentrations of the tested compounds. To measure the unspecific binding, 1 μM of propranolol was used. The incubation was terminated by rapid filtration through Whatman GF/C filters using a vacuum manifold (Millipore, Burlington, MA, USA). The filters were then washed twice with the assay buffer and placed in scintillation vials with a liquid scintillation cocktail. Radioactivity was measured in a WALLAC 1409 DSA liquid scintillation counter (Perkin Elmer, Boston, MA, USA). All the assays were made in duplicates, and the inhibitory constants (K_i_) were estimated.

### 4.4. Prophylactic Antiarrhythmic Activity in Adrenaline-, Aconitine-, and Calcium Chloride-Induced Arrhythmia

The experiments were conducted according to the method described by Szekeres and Papp [46]. The intravenous administration of adrenaline (20 μg/kg), aconitine (20 μg/kg), or calcium chloride (140 mg/kg) solution into the caudal vein of anesthetized rats (thiopental, 75 mg/kg) induced the heart rhythm disturbances. The studied compounds were injected *iv* 15 min before the arrhythmogen. The ECG was recorded during the first 2 min and in the 5th, 10th, and 15th min after the adrenaline, aconitine, or calcium chloride injection. The criterion of antiarrhythmic activity was the decrease or complete absence of extrasystoles, atrioventricular blocks, and bradycardia in the ECG recording compared with the control group (in the case of aconitine- and calcium chloride-induced arrhythmia the absence of fibrillation was also considered). The ED_50_ (a dose producing a 50% inhibition of ventricular contractions) was calculated using the method of Litchfield and Wilcoxon [47]. The tested compounds were administered at a dose of 5 mg/kg, and we gradually decreased the dose by half until the disappearance of antiarrhythmic activity.

### 4.5. Therapeutic Antiarrhythmic Activity in Adrenaline-Induced Arrhythmia

The experiments were conducted according to the method described by Szekeres and Papp [46]. The heart rhythm disturbances were evoked by intravenous administration of adrenaline (20 µg/kg) to anesthetized rats (thiopental, 75 mg/kg). The tested compounds were administered at a dose of 5 mg/kg *iv* immediately after the injection of adrenaline. The ECG was recorded during the first 2 min and in the 5th, 10th, and 15th min after the arrhythmogen injection. The criterion of antiarrhythmic activity was the decrease or complete absence of extrasystoles, atrioventricular blocks, and bradycardia in the ECG recording in comparison with the control group [20].

### 4.6. Effect on a Normal Electrocardiogram in Rats

To investigate how the tested compounds affect the normal electrocardiogram (ECG), we used Aspel ASCARD apparatus (standard II lead, with the tape speed 50 mm/s and voltage calibration 1 mV = 1 cm) for ECG measurements. Normotensive rats were anesthetized with thiopental (75 mg/kg *ip*). The ECG was recorded prior to and in the 5th, 10th, and 15th min after intravenous administration of the tested compounds. The effect on PQ, QT_c_ interval, QRS complex, and heart rate was evaluated. Bazzett’s formula, QT_c_ = QT/√RR, was used to calculate QT_c_ [22]. The compounds were administered at the highest tested antiarrhythmic dose, i.e., 5 mg/kg.

### 4.7. ECG Waveform Analysis

Automated ECG waveform analysis was performed using Eleven Maze software version 0.1 from Eleven Products Sp. z o.o. (Krakow, Poland) which uses artificial intelligence neural network-based models (e.g., [48]) and statistical and mixture modeling features of ECG signals [49]. The software, with expert supervision, recognized and classified fibrillations, extrasystoles, bradycardia, and blocks. It also calculated PQ, QRS, QTc, QT, and rate parameters.

### 4.8. Influence on Blood Pressure in Normotensive Rats

In the thiopental-anesthetized normotensive rats, the right carotid artery was cannulated with a polyethylene tube filled with heparin solution to allow pressure measurements using a Datamax apparatus (Columbus Instruments, Columbus, OH, USA). After 15 min of the stabilization period, the tested compounds were administered intravenously, and their effect on blood pressure was measured. The tested compounds were administered at a dose of 5 mg/kg, and we gradually decreased the dose by half until the disappearance of hypotensive activity.

### 4.9. Influence on Blood Vasopressor Response in Rats

To establish whether the hypotensive effect of tested compounds is a result of their α_1_-adrenolytic properties, we checked their effect on the pressor response to methoxamine (150 µg/kg). Normotensive rats were anesthetized with thiopental (75 mg/kg *ip*). The right carotid artery was cannulated with a polyethylene tube filled with heparin solution to allow blood pressure measurements using a Datamax apparatus (Columbus Instruments, Columbus, OH, USA). After a 15-min stabilization period, we measured the pressor response to methoxamine before (control) and 5 min after the administration of the tested compounds. The tested compounds were administered at the lowest dose that decreased both systolic and diastolic blood pressure, i.e., 1 mg/kg.

### 4.10. Statistical Analysis

The number of animals in groups was based on our previous studies [35]. Results are presented as either means ± SD or as a percentage of occurrence of specific events (extrasystoles, fibrillations, bradycardias, mortality). In our analysis, we used one-way repeated measures or two-way ANOVA followed by Dunnet’s or Bonferroni post hoc.

## 5. Conclusions

In this study, we demonstrated that pyrrolidine-2-one derivatives, S-61 and S-73, possess potent prophylactic and therapeutic antiarrhythmic activity in adrenaline-induced arrhythmia, probably mediated via α_1_-adrenoceptors. It is worth mentioning that neither S-61 nor S-73 exerted proarrhythmic potential. Moreover, both compounds decreased blood pressure in normotensive rats. Our study indicates that pyrrolidine-2-one derivatives can be promising compounds in preventing and treating arrhythmia. However, further studies are needed to characterize better their pharmacological properties and safety profile.

## Figures and Tables

**Table 1:** The affinity of S-61 and S-73 for adrenergic β_1_ receptors.

Treatment	Adrenergic Receptors—*p*K_i_
	β_1_
S-61	nc
S-73	nc
Propranolol	7.82

Data are represented as *p*K_i_, that is, −logK_i_ and expressed as means from three independent experiments performed in duplicates. Inhibition constants (K_i_) were calculated according to the equation of Cheng and Prusoff [21]. Radioligand binding was performed using rat cortex tissue. The affinity values were determined using [^3^H]-CGP-12177; nc—not calculable.

**Table 2:** The prophylactic antiarrhythmic activity of pyrrolidin-2-one derivatives in adrenaline-, calcium chloride-, and aconitine-induced arrhythmia models.

Treatment	Dose (mg/kg)	Fibrillations (%)	Extrasystoles (%)	Bradycardia (%)	Blocks (%)	Mortality (%)
Adrenaline-Induced Arrhythmia						
Control	-	-	100	100	100	100
S-61	0.5	-	50	33	33	0
	0.25	-	83	17	17	0
	0.125	-	100	100	100	33
S-73	0.5	-	67	33	67	33
	0.25	-	67	67	83	33
	0.125	-	100	67	83	50
Calcium Chloride-Induced Arrhythmia						
Control	-	100	100	100	100	100
S-61	5	100	67	100	100	100
S-73	5	83	100	100	100	100
Aconitine-Induced Arrhythmia						
Control	-	100	100	100	100	100
S-61	5	100	100	100	100	100
S-73	5	67	100	100	83	100

The tested compounds were administered intravenously (*iv*) 15 min before the experiment. The control group received no treatment except the administration of arrhythmogen. The observation was performed for 15 min after the *iv* injection of adrenaline (20 μg/kg), calcium chloride (140 mg/kg), or aconitine (20 μg/kg), i.e., during the first 2 min, at 5th, 10th, and 15th min. Results are presented as a percentage of the occurrence of specific cardiac events (fibrillations, extrasystoles, bradycardia, blocks, mortality); n = 6 rats.

**Table 3:** The therapeutic antiarrhythmic activity of pyrrolidin-2-one derivatives in adrenaline-induced arrhythmia model.

Treatment	Dose (mg/kg)	Bradycardia (%)	Blocks (%)	Mortality (%)
Control	-	100	83	67
S-61	5	50	33	0
S-73	5	50	67	0

The tested compounds were administered intravenously (*iv*) immediately after the injection of adrenaline at a dose of 5 mg/kg. The control group received no treatment except the administration of arrhythmogen. The observation was performed for 15 min. Results are presented as a percentage of the occurrence of specific cardiac events (bradycardia, conduction blocks, mortality); n = 6 rats.

**Table 4:** The influence of S-61 and S-73 on heart rate and ECG parameters.

Treatment	Dose (mg/kg)	Parameters	Time of Observation [min]			
			0	5	10	15
S-61	5	PQ	42.89 ± 1.31	43.44 ± 2.25	41.33 ± 1.12	44.89 ± 1.50
		QRS	35.44 ± 0.98	37.11 ± 3.17	37.89 ± 1.36	36.11 ± 1.15
		QT_c_	178.2 ± 3.93	171.8 ± 6.53	178.6 ± 6.69	179.9 ± 5.05
		QT	80.57 ± 0.69	81.53 ± 4.44	82.47 ± 5.35	83.90 ± 4.24
		Rate	312.2 ± 4.39	272.0 ± 1.66 ****	279.2 ± 7.56 ****	307.0 ± 7.21
S-73	5	PQ	47.11 ± 2.14	48.33 ± 2.22	47.56 ± 1.38	48.11 ± 2.08
		QRS	29.44 ± 1.77	30.33 ± 2.30	30.67 ± 1.40	30.67 ± 1.52
		QT_c_	185.9 ± 11.35	186.4 ± 15.84	180.0 ± 18.47	180.2 ± 20.72
		QT	80.57 ± 0.69	81.53 ± 4.44	82.47 ± 5.40	83.90 ± 4.24
		Rate	320.0 ± 13.81	278.1 ± 19.31 ****	277.1 ± 7.77 ****	269.0 ± 10.95 ****

The tested compounds were administered intravenously (*iv*), and the observation was performed for 15 min post-injection. Statistical analysis: one-way repeated measures ANOVA (Dunnett’s post ho*c*), **** *p* < 0.0001; n = 6 rats. QT_c_—calculated QT interval according to Bazzett’s formula: QT_c_ = QT/√RR [22].

## Data Availability

Data is contained within the article.
